# When Neurodevelopment Meets Autoimmunity: Pemphigus Foliaceus in Rett Syndrome Expands the Clinical Spectrum—A Case Report

**DOI:** 10.3390/ijms27156862

**Published:** 2026-07-30

**Authors:** Jatinder Singh, Samiya Chishti, Shashidhar Ameenpur, Federico Fiori, Leighton McFadden, Hassan Aziz Mirza, Lovro Vidmar, Zvi Zahavi, Paramala Santosh

**Affiliations:** 1 Department of Child and Adolescent Psychiatry, Institute of Psychiatry, Psychology and Neuroscience, King’s College London, London SE5 8AF, UK; 2 Centre for Interventional Paediatric Psychopharmacology and Rare Diseases (CIPPRD), South London and Maudsley NHS Foundation Trust, London SE5 8AZ, UK; 3 Barking, Havering and Redbridge University Hospitals NHS Trust, Romford RM7 0AG, UK; 4 Institute for Genomic Literacy, 1241 Kamnik, Slovenia; 5 Geneplanet d.o.o., 1210 Ljubljana, Slovenia; 6 Myogenes Limited, Borehamwood WD6 4PJ, UK

**Keywords:** case report, autoimmune dermatological disease, Rett syndrome, pemphigus foliaceus

## Abstract

Rett syndrome (RTT, OMIM 312750) is a complex multisystem neurodevelopmental disorder. Evidence suggests that RTT may have an autoimmune component and inflammatory activation. However, the autoimmune manifestations remain poorly described. Pemphigus foliaceus is a debilitating autoimmune blistering condition caused by IgG autoantibodies that target desmoglein-1 (Dsg1), resulting in widespread skin blistering and lesions. We report a case of pemphigus foliaceus in a 20-year-old female with RTT and discuss its clinical implications. Clinical data obtained from electronic health records were extracted and reviewed. Genetic testing was performed to identify the specific *methyl-CpG-binding protein 2* (*MECP2*) mutation and on an expanded panel of 55 genes associated with pemphigus foliaceus and related blistering disorders. The individual had pemphigus foliaceus, which required immunosuppression, intravenous immunoglobulin (IVIg) therapy, and Rituximab. The disease trajectory was complicated by infections, aspiration pneumonia, and hypoxic cardiac arrest. There was progressive functional decline, and disease control was difficult to achieve, with frequent flares. Genetic testing confirmed a heterozygous pathogenic *MECP2* variant (NM_001110792.1:c.952C>T; p.(Arg318Cys)). HLA genotyping identified alleles consistent with the HLA-DRB1*04:02–HLA-DQA1*03:01–HLA-DQB1*03:02 (DR4/DQ8) haplotype. Furthermore, genetic analysis identified a heterozygous *DSG1* variant rs12967407. This study reports the first case of pemphigus foliaceus in RTT, expanding the clinical spectrum of RTT beyond its neurodevelopmental phenotype. The DR4/DQ8 haplotype, previously associated with pemphigus susceptibility, supports a background of genetic susceptibility in this individual. No causal association between RTT and pemphigus foliaceus can be inferred from this single case. Rather, this case demonstrates that a rare autoimmune disorder such as pemphigus foliaceus can co-occur with a pathogenic *MECP2* mutation. The coexistence of a genetic and autoimmune disease can result in a more complex clinical presentation and treatment course. The case further emphasises the need for increased vigilance in identifying new and emerging systemic pathology alongside RTT.

## 1. Introduction

### 1.1. Rett Syndrome as a Multisystem Disorder

Mutations in the epigenetic regulator gene *methyl-CpG-binding protein 2* (*MECP2*) most commonly cause Rett syndrome (RTT), a multisystem disorder. It usually manifests in females; however, improvements in genetic testing have also led to its identification in males [1]. Rett syndrome typically progresses through four clinical stages: an early-onset stage occurring between 6 months and 1.5 years of age (Stage I), a developmental regression stage (Stage II) usually occurring between 1.5 years and 4 years, a pseudo-stationary period (Stage III), and Stage IV, usually exemplified by motor deterioration [2]. Some staging criteria, especially those related to Stage I with an apparently normal neurodevelopmental window, may require revision, as prodromal abnormalities and atypical neurodevelopmental features can be observed prior to Stage I [3,4,5].

Although genotype likely contributes to phenotypic variability at the group level, there is marked clinical heterogeneity even in individuals with the same pathogenic *MECP2* mutations. RTT is, therefore, primarily a clinical diagnosis [6,7]. One contributor to this clinical heterogeneity is X-chromosome inactivation (XCI). Recently, miR106a has been identified as an important regulator of XCI [8]. Other factors influencing clinical variability include additional disease and epigenetically related modifiers [9,10,11,12]. Most skills in RTT are lost early in life [13]. However, atypical neurodevelopmental trajectories have been observed, including *MECP2*-related RTT without developmental regression [14] and delayed-onset forms of regression [3]. Taken together, these findings support the view of RTT as a complex multisystem disorder spanning multiple organ systems.

### 1.2. Immune Dysregulation and Autoimmunity in Rett Syndrome

Beyond neurodevelopmental heterogeneity, immune dysregulation and neuroinflammation may contribute to the pathophysiology of RTT [15,16,17]. A subclinical inflammatory state was first proposed in 2014 [18]. Further data demonstrated that increased salivary cytokines may correlate with disease severity in RTT [19]. This chronic inflammation may also extend to other organ systems in RTT, such as the respiratory system, where alveolar inflammation has been observed [20]. While it is established that brainstem immaturity can contribute to breathing dysregulation in RTT [21], the presence of inflammatory lung disease in RTT suggests that the breathing phenotype may also have an unrecognised inflammatory or dysregulated immune system component and is not solely governed by neurological dysfunction. Inflammatory manifestations like cholecystitis have been documented in RTT [22]. Repeated scarring of the gallbladder due to inflammatory processes [23] and obstruction can lead to biliary tract disease in females with RTT [24]. A dysregulated inflammasome response is thought to be a key contributor to the subclinical inflammatory response in RTT [23]. Indeed, a sustained inflammasome response has been linked to neuroinflammation [25] and neurodevelopmental disorders [26].

Chronic inflammation has been associated with autoimmunity [27], and involvement of *MECP2* in autoimmunity has been shown in primary Sjögren’s syndrome [28] and systemic lupus erythematosus [29]. Furthermore, the hypothesis that RTT may be associated with autoimmunity was proposed a decade ago [30]. Different autoimmune-related phenomena in RTT have also been described in the literature [23]. When viewed collectively, these data suggest an immune system dysregulation in RTT that might have an association with autoimmunity. However, despite this evidence, there is a paucity of literature on dermatological autoimmune manifestations in RTT.

### 1.3. Pemphigus Foliaceus—An Autoimmune Disorder

Pemphigus foliaceus forms part of a group of autoimmune blistering disorders, including pemphigus vulgaris, paraneoplastic pemphigus, and IgA pemphigus [31]. These disorders are characterised by autoantibodies that target desmosomes, which are integral components of the skin and mucous membranes. Pemphigus is a debilitating dermatological condition whose symptoms and severity can vary between individuals. Its incidence is rare, with pemphigus vulgaris being the most common subtype [31]. Pemphigus foliaceus is a rare type of pemphigus caused by IgG autoantibodies that target desmoglein-1 (Dsg1), a desmosomal cadherin transmembrane glycoprotein. This protein is important for cell adhesion and is found in the granular layer of the epidermis [31,32], with strong expression levels in the upper trunk [31]. The IgG autoantibodies cause a loss of cell-to-cell adhesion in keratinocytes, resulting in blisters in a process called acantholysis [33]. The disease is characterised by flaccid blisters and bullae that rupture easily, resulting in scaly, crusty lesions [34]. In some cases, skin lesions may result in exfoliative erythroderma, a rare, severe, and potentially life-threatening skin condition [34]. The genetic aetiology of pemphigus foliaceus is complex. Evidence suggests that it is a polygenic disorder with major histocompatibility complex (*MHC*) class II loci (i.e., human leukocyte antigen [HLA] alleles) and *DSG1* polymorphisms as risk factors for disease [35,36,37]. Specifically, the combination of *HLA* class II genes and *DSG1* polymorphisms can increase the genetic risk [37]. Other genes are also thought to confer genetic susceptibility [35].

### 1.4. Case Significance

Although associations between RTT and autoimmune interactions have been proposed, they remain rare and have not been well characterised. Dermatological autoimmune conditions remain exceptionally uncommon, and, to the best of our knowledge, pemphigus foliaceus has not been described in RTT. Against this background, we describe the first reported case of pemphigus foliaceus in a 20-year-old female with RTT. The aim of this case report is therefore to describe the clinical features and management of pemphigus foliaceus in an individual with RTT, highlighting a rare co-occurrence of these conditions. We further underscore that the presence of RTT should not alter the standard diagnostic and clinical management of pemphigus foliaceus.

## 2. Methods

This case report was structured in accordance with CARE guidelines [38].

### 2.1. Data Collection

Information for this case was obtained from electronic health records and clinical documentation from the Centre for Interventional Paediatric Psychopharmacology and Rare Diseases (CIPPRD), South London and Maudsley NHS Foundation Trust. Where appropriate, all relevant clinical data pertaining to RTT and pemphigus foliaceus were extracted and reviewed.

### 2.2. Clinical Information Extraction and Review

Clinical information (data) was extracted by the first author (J.S.). This included demographic data, clinical history of RTT and pemphigus foliaceus, diagnostic findings, and other related information. The second author (S.C.) independently verified and reviewed the clinical information to ensure accuracy. Any discrepancies in the extracted clinical information were resolved through discussion between the first and second authors. Historical clinical documentation indicated that the individual had RTT with an *MECP2* mutation at age 5 years old; however, the specific variant was not specified in the available clinical records. Repeat *MECP2* testing was therefore performed in 2026 to determine the exact *MECP2* molecular variant.

### 2.3. Clinical Global Impressions—Efficacy Index (CGI-EI)

The Clinical Global Impressions—Efficacy Index (CGI-EI) was determined from the individual’s electronic health records describing the improvement in symptoms and medication side effects over time. The CGI-EI reflects the change in symptoms and side effects of medications and has been used previously to monitor symptom change and medication side effects in RTT [39]. The CGI-EI is scored from 0.25 to 4.00. Higher scores indicate greater therapeutic benefits relative to treatment-related adverse effects. Scores near 4.00 indicate good efficacy with minimal side effects; however, a score of 0.25 suggests that the side effects outweigh clinical benefits. Finally, CGI-EI scores around 2.00 reflect a moderate balance between symptom improvement and medication tolerability.

### 2.4. Genetic Testing

Following parental consent, an oral saliva sample was obtained for genetic analysis. The sample was processed by Myogenes (Myogenes, Borehamwood, UK) and sent to My Geneplanet (Ljubljana, Slovenia) for genetic analysis. The procedure involved gDNA isolation and Whole-Genome Sequencing to a depth of 30×, using commercially available reagents and standard protocols. BWA-MEM was used to align the reads to the hg38 reference genome, and variant calling was performed with HaplotypeCaller, employing the GATK (ver. 4.6) best-practices pipeline [40,41]. Variant calling was limited to a selected gene panel: *MECP2* to confirm the molecular variant of RTT and an expanded panel of 55 genes associated with pemphigus foliaceus and other phenotypically similar skin conditions. Variants were annotated and classified according to the American College of Medical Genetics and Genomics (ACMG) pathogenicity criteria using BIAS-2015 (v3.0.0) [42] and were additionally filtered for systemic artefacts using a local database. HLA-HD was used in parallel to genotype the HLA loci. Additionally, a previously reported pemphigus foliaceus-associated common variant, the *DSG1* c.809 polymorphism (*rs12967407*) was genotyped using the same pipeline.

### 2.5. Informed Consent and Ethics Approval

This study is part of the Tailored Rett Intervention and Assessment Longitudinal (TRIAL) Data Warehouse study that received ethics approval from the London-Bromley Research Ethics Committee on 19 October 2022—substantial amendment 7 (REC reference: 15/LO/1772). Parental written consent was obtained on 4 March 2024 for the use of information for research and publication purposes. Verbal consent was also obtained on 30 March 2026 to reconfirm participation.

## 3. Case Presentation

The case describes a 20-year-old female of Turkish origin with RTT, autism spectrum disorder, and intellectual disability. She was referred to the CIPPRD in 2019 due to concerns regarding challenging behaviour, including episodes of screaming, head banging, and face hitting, as well as sleep disturbances. There is also a history of generalised anxiety disorder, and additional comorbidities include gastroesophageal reflux disease, constipation, scoliosis, hypersalivation, and astigmatism. Investigations for abnormal movements ruled out epileptiform activity. There has been increasing medical input in recent years, including the placement of a peripherally inserted central catheter (PICC line) in November 2023. She is on a complex treatment regimen, including immunosuppressive, antiseizure, psychotropic, analgesic, gastrointestinal, dermatological, and ophthalmic treatments. The individual was trialled on multiple medications for ongoing symptom management. Table 1 summarises the therapeutic benefit and treatment-emergent side effects associated with each medication, as assessed using the CGI-EI.

### 3.1. Rett Syndrome

There was a developmental delay in all her milestones with characteristic RTT hand stereotypies. She achieved independent standing and began walking around 20 months of age; however, she no longer ambulates and uses a wheelchair. Early speech development began at 12 months of age with one or two words, followed by regression to nonverbal status. She was diagnosed with RTT at about 5 years of age. In 2026, updated molecular testing identified a heterozygous pathogenic *MECP2* variant, NM_001110792.1:c.952C>T. This results in a missense change p.(Arg318Cys) (R318C) in the protein. The variant is classified as pathogenic in ClinVar (Accession: VCV000011824.93) and has been reported previously in RTT [43].

### 3.2. Pemphigus Foliaceus

Pemphigus foliaceus was diagnosed in 2024, with additional flares in 2025. In October 2025, she was admitted to the hospital with a flare of pemphigus foliaceus that caused sepsis requiring an intensive care unit (ICU) admission for venous access. While in ICU, she had an aspiration event after vomiting and a hypoxic cardiac arrest (secondary to aspiration pneumonia) with 5 min of pulseless electrical activity (PEA). Return of spontaneous circulation was achieved at 5 min.

Repeated flares of pemphigus foliaceus caused widespread blisters and lesions. The lesions were observed on her face, scalp, chest, back, armpits, groin, hands, and arms, with fewer lesions on her legs. She was managed with immunosuppression and supportive therapy (prednisolone and antihistamines), alongside topical dermatological treatments. Regular infusions of intravenous immunoglobulin (IVIg) were also administered, with five completed cycles.

**Figure 1:**
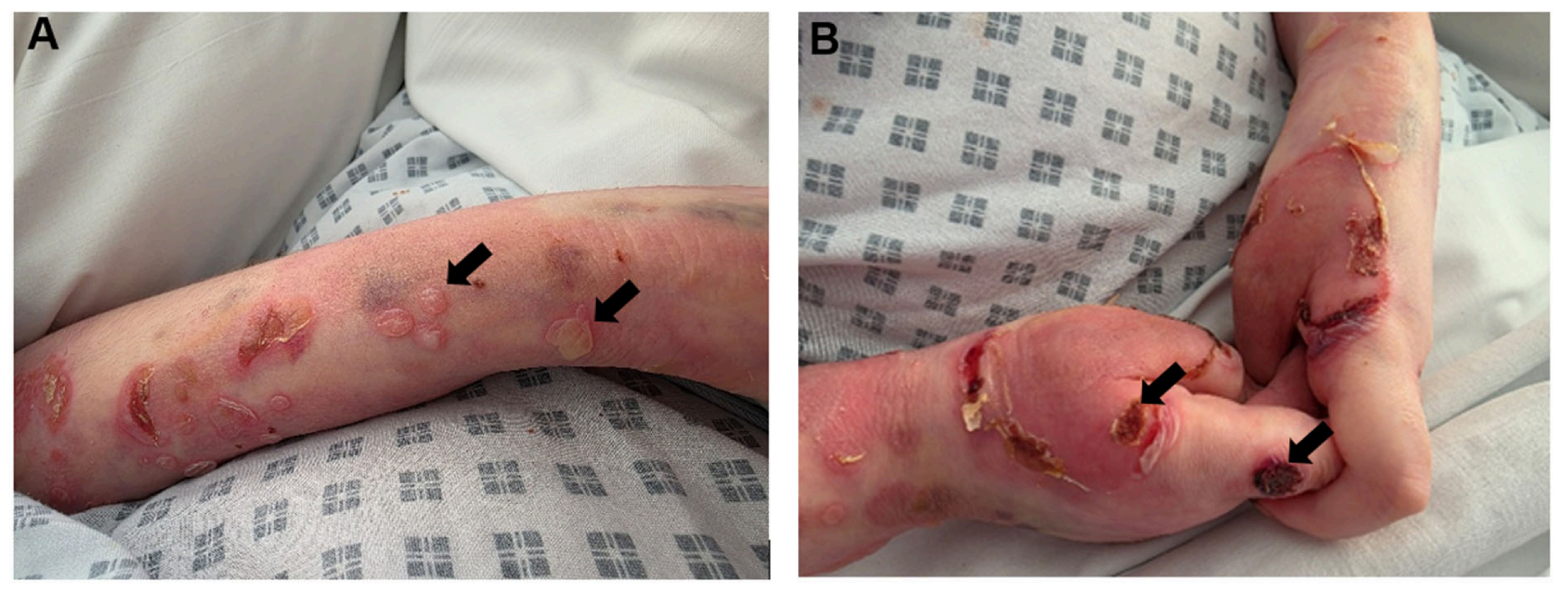
Pemphigus Foliaceus: A novel finding in a 20-year-old female with RTT. Panel (**A**) shows newly formed flaccid vesicles and bullae on the dorsal surface of the right wrist and forearm (black arrows). Panel (**B**): Crusted erosions and scabbed lesions on the dorsal surface of both hands, near the base of the thumb and index finger (arrows) (image taken in April 2026).

In 2026, she was again hospitalised due to flares of pemphigus foliaceus and associated infections. She also showed progressive physical deterioration, and admission was complicated due to the need for repeated blood transfusions. The underlying cause for the blood transfusions remains unknown at the time of reporting. During this admission, she again received IVIg. However, the infusions were notably less effective than previously observed, with recurrent blisters over her arm, face, and body, suggesting that pemphigus foliaceus had become refractory to IVIg treatment. Figure 1 shows the involvement of the hands and arms.

In May 2026, she received the first dose of Rituximab. The individual had an initial flare with the first dose of Rituximab and then got better when given a dose of IVIg. No new blisters were reported, and her existing skin lesions were noted to be healing more quickly. She will continue to be managed with Rituximab and IVIg therapy.

### 3.3. Genetic Analysis

In June 2026, high-resolution genotyping of the HLA loci was performed. This identified alleles at class I (HLA-A, -B, -C) and class II (HLA-DRB1, -DQA1, -DQB1, -DPB1) loci (Table 2).

The individual was also heterozygous for the *DSG1* variant rs12967407 (c.732C>T/c.809C>T), carrying one copy of the reported pemphigus foliaceus-associated C allele. She does not carry the homozygous C/C (809) genotype, which has been reported to confer increased risk of pemphigus foliaceus [37].

Targeted analysis of the 55-gene panel identified no pathogenic or likely-pathogenic variant in any non-HLA gene. Two heterozygous missense variants of uncertain significance, both in desmosomal plakophilin genes, were detected:

(I) PKP1, c.490C>T p.(Arg164Trp) (*rs576171894*), heterozygous; gnomAD allele frequency 4.6 × 10^−5^; classified as a variant of uncertain significance in ClinVar (VCV003889631).
(II) PKP3, c.1304G>A p.(Arg435His) (*rs777411352*), heterozygous; gnomAD allele frequency 4.6 × 10^−5^; classified as a variant of uncertain significance in ClinVar (VCV002591591).

Neither of these variants were associated with the individual’s disease. Biallelic loss-of-function of *PKP1* causes autosomal-recessive ectodermal dysplasia–skin fragility syndrome, which is not consistent with a single heterozygous missense allele, and *PKP3* is not associated with a Mendelian disorder. Both variants passed the systemic-artefact filter and were each observed in only this individual within the in-house cohort. No other rare coding variants were identified across the remaining panel genes.

## 4. Discussion

As far as we are aware, this is the first reported case of pemphigus foliaceus in RTT. The case describes a complex clinical journey of pemphigus foliaceus presenting with severe and refractory disease that required immunosuppression, IVIg therapy, and Rituximab. The CGI-EI ratings further illustrated the complexity of symptom management when treating RTT and a co-occurring autoimmune disease with multiple medications. The clinical picture was complicated by sepsis, aspiration pneumonia, and hypoxic cardiac arrest, underscoring the systemic burden of a coexisting autoimmune disease in RTT. These medical factors are particularly relevant in the context of RTT, which is already associated with an increased risk of aspiration [6,44]. Individuals with RTT also have autonomic dysregulation and impaired thermoregulatory responses, including intra-individual temperature asymmetries [45], that may further complicate the early recognition of infections. However, while RTT introduces additional medical complexity, it should not preclude escalation of care for a systemic autoimmune dermatological disease. The case reinforces the premise that the presence of RTT should not delay the diagnosis or management of an additional disorder.

Alongside a heterozygous pathogenic *MECP2* variant (NM_001110792.1:c.952C>T), genotyping also identified the presence of HLA class II haplotype ‘HLA-DRB1*04:02–HLA-DQA1*03:01–HLA-DQB1*03:02 (DR4/DQ8)’. While it is not known whether this exact haplotype is causative of pemphigus foliaceus, the HLA-DR4/DQ8 class II haplotype has been associated with increased susceptibility to pemphigus foliaceus [46,47]. In particular, the HLA-DRB1*04 alleles (including DRB1*04:02), together with HLA-DQB1*03:02 (DQ8), have been implicated as key components of this susceptibility haplotype [37,47]. When viewed more broadly, the DR4/DQ8 class II haplotypes are also associated with increased susceptibility to autoimmune diseases [48]. The individual also carries one copy of the *DSG1* rs12967407 risk allele (C/T genotype) but not the homozygous C/C genotype reported in the French population that is associated with increased genetic susceptibility to pemphigus foliaceus, including the epistatic interaction described with HLA class II DRB1*04 alleles [37]. While it has been reported that DRB1*04 carriers without the *DSG1* C/C(809) genotype are not at increased disease risk for pemphigus foliaceus, the Martel et al. (2001) [37] study was performed in a relatively small sample (*n* = 31) and therefore does not exclude a potential contribution from the heterozygous *DSG1* C/T genotype. Further work is needed to see whether the heterozygous *DSG1* C/T genotype confers any modifying risk. In summary, genotyping identified the DR4-DQ8 haplotype, which is known to confer increased genetic susceptibility to pemphigus foliaceus. The individual does not carry the high-risk homozygous genotype for *DSG1* or pathogenic/likely-pathogenic variants in other desmosomal, immune blistering or immune regulatory genes.

A key clinical observation was the aggressive nature of pemphigus foliaceus. Despite repeated therapeutic interventions, including cycles of IVIg and systemic immunosuppressive therapy, disease control was difficult to achieve prior to Rituximab therapy, with frequent flares. A recent systematic review on the treatment of pemphigus foliaceus has shown that, in many cases, treatment is refractory to conventional immunosuppressive therapy [49]. Biological therapies such as IVIg and Rituximab are increasingly used; however, response rates remain variable [49]. Given that Dsg1 is expressed in both the peripheral and central nervous systems, there is evidence of an association between pemphigus and neurological disease [33]. In parallel, some evidence indicates that RTT may involve broader immunological processes, including an autoimmune component [30]. Although these findings raise the possibility of shared immunological or autoimmune mechanisms, it is unknown whether the dysregulated immune response in RTT leads to chronic inflammatory priming that can affect the disease trajectory of pemphigus foliaceus. If there were a strong immunological association between RTT and pemphigus foliaceus, a greater number of cases might be observed. This single case report does not imply a causal relationship between RTT and pemphigus foliaceus. The genetic analysis identified a co-occurring HLA-DR4/DQ8 class II haplotype, which is associated with increased susceptibility to pemphigus foliaceus. Further work would be required to determine whether the hypothesis regarding the underlying immune dysregulation in RTT may have contributed to the severity of pemphigus foliaceus.

This case report has limitations. First, this is a single-case report; therefore, no inferences about causality between RTT and pemphigus foliaceus can be made. Second, incomplete documentation on pemphigus foliaceus, including the lack of diagnostic testing and disease markers such as immunofluorescence, histopathology, and autoantibody titres, limited detailed analysis of disease progression. Third, while pemphigus foliaceus is currently being managed using biological therapies such as IVIg and Rituximab, the long-term outcome remains unknown. Fourth, the CGI-EI scores were obtained retrospectively from the electronic health records. As the completeness of the records may have varied across clinical encounters, the information used to generate the CGI-EI scores may not have captured the individual’s true clinical presentation with regard to symptom improvement and side effects. Finally, the HLA/DSG1 associations were assessed without a comparator cohort, limiting inferences regarding their contributions to pemphigus foliaceus disease susceptibility.

## 5. Conclusions

This single case emphasises the importance of increased awareness of autoimmune pathology co-occurring alongside RTT and suggests the need to further evaluate the clinical spectrum of RTT beyond the neurodevelopmental phenotype. It also underscores the notion that having RTT does not preclude the development of an additional significant pathology [9]. Genotyping identified an HLA-DRB1*04:02–HLA-DQA1*03:01–HLA-DQB1*03:02 (DR4/DQ8) haplotype demonstrating the individual’s increased genetic susceptibility to pemphigus foliaceus. This case also highlights the risk of diagnostic overshadowing. While the diagnosis of RTT adds complexity, it should not delay timely assessment or the escalation of care for pemphigus foliaceus. As illustrated in this case, “*when neurodevelopment meets autoimmunity*” represents an emerging clinical intersection that warrants further investigation.

## Figures and Tables

**Table 1:** Clinical Global Impressions—Efficacy Index (CGI-EI) ratings for medications used for ongoing symptom management.

Medication	Symptom Improvement	Treatment-Emergent Side Effects	CGI-EI
Aripiprazole	MODERATE	NONE	3.00
Sertraline	MINIMAL	NONE	2.00
Mirtazapine	MODERATE	NONE	3.00
Diazepam	MODERATE	NONE	3.00
Promethazine	MODERATE	NONE	3.00
Trihexyphenidyl	MINIMAL	NONE	2.00
Dexamethasone	MODERATE	SEVERE	0.75
Hyoscine butyl bromide	MODERATE	NONE	3.00
Mefenamic acid	MINIMAL	NONE	2.00
Morphine solution	MINIMAL	NONE	2.00
Paracetamol	MINIMAL	NONE	2.00
Prednisolone	MODERATE	SEVERE	0.75
IVIg	MODERATE	NONE	3.00
Rituximab	MODERATE	MILD	1.50

Abbreviations: CGI-EI, Clinical Global Impressions—Efficacy Index; IVIg, intravenous immunoglobulin.

**Table 2:** HLA class I (A, B, C) and class II (DRB1, DQA1, DQB1, DPB1) genotyping.

Locus	Allele 1	Allele 2
HLA-A	*02:01	*02:01
HLA-B	*51:01	*44:27
HLA-C	*07:04	*15:13
HLA-DRB1	*16:15	*04:02:01
HLA-DQA1	*01:02:02	*03:01:01
HLA-DQB1	*05:02:01	*03:02:01
HLA-DPB1	*05:01:01	*04:01:01

* indicates the HLA allele designation; Abbreviations: HLA, human leukocyte antigen.

## Data Availability

All the data used for this case report is included in the manuscript.
